# Social inequalities in SARS-CoV-2 infection in high income countries: highlighting the need for an intersectional perspective in quantitative research

**DOI:** 10.3389/fpubh.2025.1642407

**Published:** 2025-07-31

**Authors:** Ritu Rani, Nathalie Bajos, Émilie Counil

**Affiliations:** ^1^Institut de Recherche Interdisciplinaire sur les enjeux Sociaux - Sciences Sociales, Politique, Santé, IRIS (UMR 8156 CNRS - EHESS - U997 INSERM), Aubervilliers, France; ^2^Institut National d'études Démographiques/ French Institute for Demographic Studies (INED), Aubervilliers, France

**Keywords:** social inequalities, intersectionality, quantitative analyses, COVID-19, France

## Abstract

**Objective:**

COVID-19 has highlighted significant social disparities in health across the world. The main purpose of this mini review is to synthesize important findings about social inequalities in infection risk and highlight the gaps in current research from an intersectional perspective.

**Method:**

This mini literature review builds on peer-reviewed quantitative studies conducted in high-income countries, with a particular focus on France. It outlines key findings on inequalities across gender, socioeconomic status and ethnoracial background, summarizes methodological approaches, and locates research gaps.

**Results:**

Evidence on gender inequalities in infection risk remain mixed. Most studies reported increased risks among marginalized groups, including racialized minorities and socioeconomically disadvantaged populations. Multivariate regression models were commonly used to estimate infection risk while adjusting for individual and contextual factors in an “all other things equal” perspective. However, no study applied an intersectional framework, limiting comprehensive understanding of the intricate social dynamics of infection disparities. Some studies instead explored intersectional inequalities for outcomes like mortality and mental health during the COVID crisis. For example, a UK study found worse mental health among sexual minorities, females, and younger individuals, and revealed hidden inequalities in social groups combining privilege and disadvantage.

**Conclusion:**

Studies performed on COVID-19 outcomes other than infection do emphasize the added value of incorporating an intersectional perspective into quantitative approaches. Through the use of this framework, future research may guide the development of more targeted and equitable public health interventions to prevent the spread of similar airborne viruses across populations.

## 1 Introduction

The COVID-19 pandemic has not only highlighted the importance of infectious diseases in global health, but has also exacerbated existing social inequalities (1–3). Studies from multiple countries have reported higher COVID-19 risk among the socially deprived individuals (4–7). Across Europe, low-income groups and racialized minorities have been found particularly affected during the pandemic (8). In the United States, higher socioeconomic status (SES) was linked with earlier SARS-CoV-2 infection, while later on, higher COVID-19 incidence was found in lower SES group (9). In the United Kingdom, Black and South Asian populations were at greater risk of infection, due to overcrowded housing, and front-line occupational exposure (10). Similarly, in France, marginalized populations, including racialized minorities and those living in the most deprived areas experienced disproportionately high infection rates, alongside healthcare professionals (11–13). These inequalities were likely due to occupational exposure under lockdown measures, and overcrowded living conditions (1, 11). However, the social pattern of infection has changed over place and time, and the underlying mechanisms are yet not fully understood.

Explaining these complex disparities necessitates a research framework that goes beyond single-axis analyses and considers the intersecting influence of diverse social mechanisms. The concept of intersectionality offers such framework to understand how multiple social relations (e.g., race, ethnicity, gender, class) intersect at the micro level of individual experience and reflect broader systems of privilege and oppression at the macro, social-structural level (e.g., racism, sexism, classism) (14, 15). Scholars have advanced contemporary understandings of intersectionality by explaining how multiple forms of oppression interlock, creating patterns different from singular forms of discrimination or their mere addition. They emphasized that deep-rooted systems of power and oppression perpetuate domination, privilege, and persistent inequality (15–17).

This research framework has been widely applied in qualitative studies to examine social inequalities; however, its integration into quantitative health research is more recent (18). Likewise, adopting an intersectional approach is essential for understanding and addressing inequalities in the context of the COVID-19 pandemic (19). Although a few studies attempted to do so for other COVID-19 outcomes such as vaccine uptake (20), mortality (21), and mental health (22, 23), similar analyses for the risk of infection remains, to our knowledge, largely underexplored.

As a mini review this paper followed a traditional or narrative literature review approach (19, 24–26), which allows flexibility in exploring and synthesizing existing research on a rather large topic. We provide an overview of the existing quantitative evidence by conducting a search of peer-reviewed empirical studies examining social inequalities in COVID-19 infection in high income countries, with a particular focus on France. To identify relevant articles we searched Google Scholar and PubMed using different combinations of the keywords divided into four groups: (1) COVID-19 related terms (“COVID-19,” “SARS-CoV-2 infection,” “Seroprevalence of SARS-CoV-2”); (2) intersectionality related (“intersectionality,” “intersectional”); (3) social dimensions (“social inequalities,” “socioeconomic inequalities,” “gender,” “race,” “ethnicity,” “class,” “social class,” occupational class”); and (4) geographic scope (“high income countries,” “France”). At least one term from each keyword group was consistently included in the search strategy, for example: “intersectionality AND gender AND race AND class AND COVID-19 AND France.” We also identified articles using a snowball search method by reviewing the reference lists of studies retrieved from the initial keyword search. We included both original research and review articles published in English and French, between 2020 to September 2024 that were relevant for our research questions. Firstly, we summarize key findings about inequalities across different social groups, then we report on common methodological approaches, and finally, we identify research gaps and needs regarding the adoption of an intersectional lens.

## 2 Social inequalities in SARS-CoV-2 infection

### 2.1 Gender inequalities

The COVID-19 pandemic has highlighted gender differences in infection rates across high-income countries, although the evidence is mixed. SARS-CoV-2 infection in various studies was measured using different outcomes such as clinically confirmed cases, symptom-based assessments, PCR tests, antigen tests, and antibody (serological) tests. Most of the population-based studies investigating SARS-CoV-2 seroprevalence did not report significant gender disparities, such as in Spain (27), Switzerland (28), or the United States (29). The pattern observed in these single studies was confirmed in larger reviews and meta-analyses. A review of seroprevalence studies found no significant gender differences between men and women (30). Further, a systematic review and meta-analysis, which included 968 studies from 74 countries, also reported no difference in seroprevalence between genders, globally (31).

However, some single studies reported significantly higher risk of infection among women than men. For instance, in Italy, seroprevalence was significantly higher in women (32); and a German study using epidemiological modeling also found that working age women were at higher risk of infection compared to men (33). Further, a study from Canada analyzed sex-disaggregated COVID-19 data and identified a gender peak effect, where women’s share of infections consistently increased and exceeded men’s during each wave’s peak. This trend was largely explained by women’s higher representation in care work, as reflected in occupation and age variables (34).

Meanwhile, some seroprevalence studies from the US reported higher rates of SARS-CoV-2 antibodies in men compared to women (35, 36). Similarly, findings from a meta-analysis of 61 studies involving 59,254 patients also showed that men had a higher COVID-19 incidence compared to women (37). Moreover, the current body of literature suggests that men were more likely to experience severe complications and death (38).

In France, most of the studies were based on data from the French EpiCoV (Epidémiologie et Conditions de Vie sous le COVID-19) study (May 2020–Dec 2022), a large prospective cohort that captures both the epidemiological and social dimensions of the pandemic. The cohort included individuals aged 15 and older (*N* = 134,391) as of 2020, residing in mainland France as well as in the overseas departments of Martinique, Guadeloupe, and Réunion, excluding those living in prisons or nursing homes. Using this data, a study conducted during the early stage of the pandemic revealed that women were more likely to report symptoms such as anosmia and ageusia during and right after the first pandemic’s peak (39). Another study examining trends in social exposure to SARS-CoV-2 infection in France found that, as of May 2020, seroprevalence was slightly higher among women than men. However, by November 2020, no significant difference in seroprevalence was observed (13). Another multicohort study conducted across three regions in France (Ile-de-France, Grand Est, and Nouvelle-Aquitaine) found a positive association between seropositivity and female gender only in the Nouvelle-Aquitaine region (40). These apparently conflicting results are likely reflecting complex and dynamic interplays of social, demographic and occupational factors, such as gendered family roles and labor market segregation, that further need to be better understood.

### 2.2 Socioeconomic inequalities

The role of socioeconomic inequalities in shaping SARS-CoV-2 infection risk has been extensively examined in previous research at both the individual and area levels. At the individual level, indicators such as income, education and occupational status have consistently emerged as key factors in explaining infection risk. Studies from Spain, the United Kingdom, and South Korea indicated that individuals with lower income levels faced a greater risk of infection, despite differences in income classification across contexts (41–43). Similarly, studies from Portugal and United Kingdom identified lower educational attainment as a risk factor of SARS-CoV-2 infection (42, 44, 45). However, studies have shown inconsistent association between occupational class and the risk of infection across countries. A study in the United Kingdom reported higher risk among lower occupational classes (such as technical workers, personal service occupations, and machine operatives) compared to higher class (managers and senior officers) (46). In contrast, a study from Switzerland found no significant association between seropositivity and occupational class (47), while research from Germany showed a higher infection risk among individuals in higher occupational classes (48). Further, occupation also played a critical role, with frontline workers, particularly those in essential sectors such as healthcare, logistics, retail, and public transportation reported higher infection rates (49, 50). These findings align with a scoping review, primarily from the United Statesand United Kingdom, which reported that socioeconomically disadvantaged groups faced significantly higher infection risks compared to their more privileged counterparts (51). Another systematic review further confirmed this pattern, consistently reporting a positive association between lower socioeconomic status, and increased infection risk across all examined studies (52).

Some ecological studies examined the association between area-level socioeconomic deprivation indices and SARS-CoV-2 infection rates. Studies from Switzerland found that individuals residing in neighborhoods with low socioeconomic status were more likely to test positive (53, 54). In addition, a scoping review of studies from high income countries examined the temporal dynamics of socioeconomic inequalities in COVID outcomes and found that a majority of the studies reported stable or widening socioeconomic inequalities in incidence, with disadvantaged populations being the most affected. The review also highlighted temporal shifts, where higher infection rates were observed among affluent populations during early waves, followed by a crossover to higher rates in socioeconomically disadvantaged populations (4). Similarly, studies from Spain, the US, and Germany analyzed area-based deprivation indices in relation to incidence rates, identifying crossover dynamics over time (9, 41, 55).

Early studies from France revealed that patient-facing and public-facing workers, including those in healthcare, social services, retail, and manufacturing, likely faced higher work-related exposure due to limited telework feasibility (56). Further, a study found that SARS-CoV-2 infections declined more significantly during lockdown among the privileged class compared to the working class (11). Another study showed that healthcare workers reported higher seroprevalence compared to those in other occupations during the first and second waves of the pandemic (13). In regards to income and education, findings from the same study showed that seroprevalence followed a complex pattern, being highest among individuals in both the highest and lowest income deciles and lowest among those with the least education. This was partly explained by high exposure among healthcare workers and deprived populations in overcrowded urban housing. Further, a study examined the effect of area-based social deprivation on SARS-CoV-2 infection and found a positive social gradient between deprivation and the likelihood of testing positive for COVID, with individuals in the most deprived areas facing the highest risk (12).

### 2.3 Ethnoracial inequalities

Evidence indicate that the risk of infection has been unevenly distributed across ethno-racial groups (6, 7, 57). A study from the US found no significant association between higher proportion of Asian or Black residents and an increased risk SARS-CoV-2 infection (58). Another study showed a higher risk of testing positive among Non-Hispanic Black and Hispanic individuals compared to White individuals (59). The UK Biobank study found higher COVID-19 positivity among Black and South Asian individuals, with Pakistani ethnicity at highest risk within the South Asian group (45). Further, a systematic review by, comprising a majority of studies from the US, confirmed that racialized minorities faced a higher risk of SARS-CoV-2 infection and were more likely to test positive than white individuals (6). These findings were also supported by a global meta-analysis on seroprevalence which revealed significantly higher rates among Black, Asian, Indigenous, and other groups compared to Caucasian individuals (31). Similarly, another recent meta-analysis of 77 studies involving over 200 million participants found that, compared to White majority populations, the risk of testing positive for COVID-19 (active infection) was significantly higher among individuals from Black, South Asian, Mixed, and Other ethnic groups. Furthermore, the analysis highlighted that Black, Hispanic, and South Asian people were also more likely to be seropositive (60).

In France, race-related data is not collected due to legal restrictions; however, studies have determined ethnoracial status based on migratory status. Research has confirmed that racialized minorities have faced disproportionately high risks of SARS-CoV-2 infection. A study based on the EPICoV cohort found that racialized minorities faced a higher risk of anosmia/ageusia during and after the pandemic’s peak, accumulating more exposure risk factors than the mainstream population (39). Another study found that Non-European immigrants in France faced higher exposure to COVID-19 risk factors, in spite of higher compliance to protective measures (61). Further, a serology study from the same cohort found that non-European immigrants had twice the seroprevalence of the native population, largely due to residential density and household size. In contrast, immigrants from European countries had seroprevalence rates similar to the native population. These findings highlight the effect of spatial segregation on immigrant populations from low- and middle-income countries (62). Moreover, results showed that the seroprevalence among second-generation immigrants from outside Europe increased from 5.9 to 14.4% from the first to second wave. This group remained significantly associated with seropositivity in second wave, even after adjusting for contextual and individual variables (13).

## 3 Quantitative methodological approaches

The existing literature provided valuable insights into social inequalities in the risk of SARS-CoV-2 infection. However, it was predominantly focused on unidimensional analyses examining social determinants such as gender, race/ethnicity, income or education in isolation. These studies employed a variety of statistical methods, ranging from descriptive analyses to advanced modeling techniques, to explore the effects of different types of power relations on infection risk. Many studies begin with descriptive analyses to summarize infection rates across different socio-demographic groups. For example, studies often compared infection rates/seroprevalence by sex, income levels, occupations, or geographic regions, providing a baseline understanding of disparities (29, 39, 40). Further, it was noted that in a majority of studies, multivariate analyses such as linear, logistic and multinomial regression models were commonly used to examine the association between social determinants and the risk of infection, net of other factors (13, 28, 32, 40, 58, 59, 63–65). These models estimated the likelihood of infection based on explanatory variables, both individual and contextual, such as age, sex/gender, income, education, housing conditions, migration status, comorbidities, occupational exposure, region/state, etc.

Some studies also included stratified and interaction analyses that provided additional insights into the variability of risks across different subgroups (28, 29, 61, 62, 65, 66). Furthermore, more advanced methods such as mediation analyses, Poisson regression model, Cox proportion hazard model, negative binomial and machine learning models has also been applied in a few studies (12, 58, 66–70). However, none of the existing studies incorporated an intersectional framework, often overlooking the compounded and intersecting effects of multiple social positions, limiting an in-depth understanding of the mechanisms underlying inequalities in infection risk.

To the best of our knowledge, only a few studies from the United States, United Kingdom and Europe have incorporated intersectionality for examining inequalities in the context of the pandemic focusing on outcomes such as mortality, mental health or vaccination (20–23, 71, 72). However, no such evidence was found from France. Using an intersectional approach in the analyses, some of these studies highlighted how socially constructed categories shaped by structural power relations, intersect to produce intricate layers of advantage and disadvantage. For example, the study by Morales (71) conducted in the US, demonstrated how gender intersected with individuals’ socioeconomic status (measured by education, household income and employment status) to influence COVID-19 vaccine hesitancy. They further found that poverty and employment influenced vaccine hesitancy among women but not men (71). However, one of the key limitations of the stratification is that it does not fully capture the complex interactions between multiple social dimensions as they are experienced within different subgroups, which can influence study outcomes (73). In a study from Sweden (20), researchers examined sociodemographic disparities in COVID-19 vaccination uptake using national register data. Logistic regression models were applied, along with an intersectional approach that included multiple cross-classified subgroups. The intersectional variable was constructed by combining categories of age, sex, income, country of birth, and occupational status, resulting in 72 strata. The findings showed that non-vaccinated individuals were more likely to be younger, male, have lower income, be unemployed, or born outside Sweden. Vaccine coverage ranged from 32% to 96% across intersectional strata/subgroups. These findings highlighted that focusing on single sociodemographic factors and group averages, without accounting for differences/heterogeneity within groups, may obscure important variations likely explained by complex social mechanisms (20).

Further, a study examined how intersecting demographic and socioeconomic factors shaped health-related quality of life (HRQoL) and mental health in children and adolescents in Geneva, Switzerland during the pandemic (22). The study applied Multilevel analysis of individual heterogeneity and discriminatory accuracy (MAIHDA) model by nesting individuals within 48 intersectional social strata based on sex, age, immigrant background, parental education, and financial hardship in Bayesian multilevel logistic models. Mental health was assessed using items from the Pediatric Global Health (PGH-7) and the Strengths and Difficulties Questionnaire (SDQ), while health-related quality of life (HRQoL) was measured using the short-form version of the Pediatric Quality of Life Inventory (PedsQL). The outcome variables of the study were poor HRQoL, poor parent-reported mental health and mental health difficulties. The range of the predicted probability for poor HRQoL varied between 3.4% (for 6–11 years old Swiss girls, with highly educated parents and no financial problems) to 34.6% (for 12–17 years old non-Swiss girls with highly educated parents and financial problems). The analyses highlighted those strata involving adolescents and financial problems consistently showed worse HRQoL. Whereas, in contrast, predicted frequency of mental health difficulties (based on the SDQ score) range varied less across groups (4.4–6.5%). Therefore, the intersectional analysis revealed diverse and outcome-specific social patterns in HRQoL and mental health and suggest that post-pandemic efforts to reduce HRQoL inequities should focus on adolescents from financially disadvantaged families, while mental health interventions should broadly support all children and adolescents (22).

A similar kind of study from the United Kingdom aimed to examine mental health inequalities among young adults during the pandemic (23). The study assessed mental health using measures of anxiety, depression, loneliness, and life satisfaction and formed intersectional strata using categories tied to social power, including age, sex, race/ethnicity, sexual orientation, and socioeconomic position. Using the MAIHDA method (23), it explored patterns of mental health measures across multiple intersecting positions and examined whether these intersections revealed effects beyond those attributable to any single position considered separately. The analysis revealed significant mental health inequalities across different intersectional strata. Much of these inequalities were driven by the additive effects of the variables used to define the intersections, with some of the largest gaps associated with sexual orientation, followed by cohort/generation, and birth sex with sexual minorities, females, and younger people (in their teens/20s) showing worse levels. Further, intersectional effects were reported mostly in intersections defined by the combinations of marginalized and privileged social positions (e.g., lower than expected life satisfaction was observed in South Asian men in their 30s from a sexual minority and disadvantaged childhood social class, followed by Black heterosexual men in their 30s from an advantaged childhood social class) (23). These sub-groups would likely have remained invisible in a fully categorical approach. Compared to the classical approaches, the results of the study make significant contribution to the literature by highlighting those inequalities are not confined to groups with exclusively advantaged or disadvantaged positions and may vary depending on the context. These intersectional effects reflect the influence of marginalization and/or privilege shaped by the interlocking systems of oppression in the studied outcomes (74) and would have been made difficult to unveil otherwise.

## 4 The need for an intersectional lens

Intersectional analyses challenge the notion that inequalities are confined solely to groups with uniformly advantaged or disadvantaged positions. Instead, they emphasize that such disparities are contingent on specific social contexts and interactions. Moreover, such analyses provide a detailed understanding of intersections that might remain undetected while using classical approaches, and provide estimates of the heterogeneity within those intersections. This highlights the importance of Hankivsky’s theorization of intersectionality that the significance of specific combinations of social (power) relations might be better revealed inductively during the research process rather than predetermined (75). The examples (20, 22, 23) pointed above and their strong conceptual foundations highlight the crucial importance of including an intersectional lens while analyzing social inequalities in the risk of SARS-CoV-2 infection. This is likely to provide a critical framework for structuring research questions and hypotheses, emphasizing the need to move beyond assumptions of homogeneity across intersecting social categories, and help to solve enigmas of inconsistent results across studies conducted in apparently similar contexts, such as those we reported about gender considered in isolation.

In spite of its promises, there are some challenges attached to the implementation the framework in empirical studies. Researchers already working within this paradigm have highlighted the lack of clear guidelines for quantitative approaches to effectively capture the complexity of intersecting social positions (14, 76). However, recent review papers have made significant progress in addressing this gap and laid the foundation by discussing theoretical and analytical approaches that are well-suited for studying intersectionality in quantitative health research (18, 73, 77, 78). For instance, researchers emphasized the importance of including intersectional group measures beyond sex/gender and race, such as sexual orientation, disability, religion, nativity, and immigration status, in order to capture a broader spectrum of social inequalities. Further, it is suggested to explicitly define the intersectional positions analyzed, their relation to social power, and to ensure alignment between theoretical approaches, methods, and interpretations. The inclusion of McCall’s intersectionality frameworks- anticategorical (deconstructing traditional categories), intracategorical (focusing on underexplored intersections), and intercategorical (using existing categories to study inequality) was recommended, with an emphasis on their explicit application and clear justification for their use (79). Moreover, in this context, Public Health Agency of Canada has also provided a checklist to strengthen the integration of intersectionality theory in quantitative health inequality analyses (78).

Another challenge, specific to the risk of SARS-CoV-2 infection and also a potential limitation of this review, is the likely complex dynamics over place and time, particularly in relation to the introduction of vaccination. The timing of vaccine rollout varied across countries and likely played an important role in shaping inequalities. While we aimed to include papers covering the pre-vaccination phase, it is possible that some studies may have combined data from pre and post-vaccination periods. This may complicate the interpretation of infection patterns and the comparability of findings across studies. Future research should account for these temporal shifts to better capture the structural drivers of infection risk.

In the French context, the lack of intersectional research on SARS-CoV-2 infection risk is particularly compelling, given the evidence of significant social inequalities during the pandemic. As discussed above, studies have documented higher infection risks among low-income people, and racialized minorities but no significant gender difference, yet these analyses were unidimensional. Incorporating an intersectional lens by accounting for multiple social dimensions would provide a more holistic understanding of these inequalities and their dynamics. In line with previous work (19, 80, 81), supporting the adoption of an intersectional perspective in analyzing the effects of social inequalities related to the COVID-19 crisis, such approaches are also crucial for informing policy responses to effectively tackle future pandemics. To address this gap, future research could use data from the French EpiCoV cohort which offers strong potential to analyze and uncover new insights into social inequalities and serve as a valuable foundation for studies in other high-income countries.
